# Digital Implant Dentistry: The Past, the Present, the Future

**DOI:** 10.1111/jre.70139

**Published:** 2026-07-06

**Authors:** Tim Joda, Jason Lee, Kevser Pala, Jessica Poon, Ronald E. Jung, German O. Gallucci

**Affiliations:** ^1^ Clinic of Reconstructive Dentistry, Center for Dental Medicine University of Zurich Zurich Switzerland; ^2^ Department of Reconstructive Dentistry, University Center for Dental Medicine Basel University of Basel Basel Switzerland; ^3^ Department of Restorative Dentistry and Biomaterials Science, Harvard School of Dental Medicine Harvard University Boston Massachusetts USA

**Keywords:** computer‐aided design/computer‐aided manufacturing (CAD/CAM), computer‐assisted implant surgery (CAIS), data acquisition, dental implant, intraoral scanning (IOS), personalized therapy

## Abstract

Digital implant dentistry has evolved from the analogue fundamentals of osseointegration to a highly data‐driven discipline that integrates advanced imaging, virtual planning, guided surgery, and computer‐aided prosthetics. The past was characterized by two‐dimensional diagnostics, freehand implant placement, and conventional impressions, which—despite their limitations—enabled pioneering therapeutic concepts. Today, cone‐beam computed tomography (CBCT), intraoral scanning (IOS), and CAD/CAM production represent disruptive technologies that have irreversibly transformed diagnostics, workflows, and prosthetic outcomes. Computer‐assisted implant surgery (CAIS), both static and dynamic, has enhanced precision and safety, while digital impressions and monolithic restorations have streamlined implant prosthetics. Digital monitoring tools now allow longitudinal assessment of peri‐implant tissues, occlusion, and wear of restorations. Looking ahead, artificial intelligence (AI) will increasingly support diagnostics and planning, while robotics may redefine surgical execution, and intraoral sensors promise personalized preventive monitoring during maintenance. The integration of all digital records into a comprehensive “digital twin” of the patient points toward a future of predictive, patient‐specific implant therapy. Critical discussion remains essential; while digital technologies offer standardization and reproducibility, they also raise economic, educational, and ethical challenges, including investment, accessibility, and data security. Digital implant therapy enhances predictability and patient experience. Finally, the digital transformation of implant dentistry is not an end in itself, but a powerful enabler–reshaping workflows, augmenting clinical expertise, and opening the door to personalized implant care.

## Graphical abstract

This narrative review examines the evolution of digital implant dentistry across the entire clinical workflow, comparing historical and contemporary approaches at each stage. Particular emphasis is placed on distinguishing truly disruptive innovations from more gradual technological advances, while also critically discussing emerging developments.

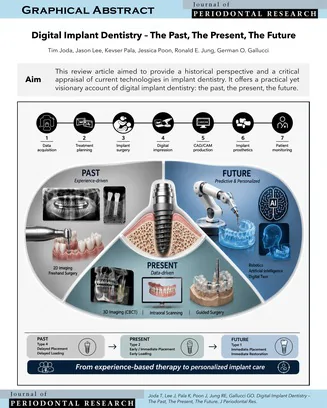

## 1 Introduction

Dental implantology has undergone a remarkable transformation since the pioneering work of Per‐Ingvar Brånemark in the 1980s established osseointegration as the biological foundation for predictable tooth replacement [1]. Over the past four decades, the field has evolved from a surgically dominated discipline into a highly interdisciplinary specialty, where prosthetic, surgical, and digital considerations converge to create patient‐centered treatment concepts [2, 3]. In parallel with this clinical development, the rapid advancement of digital technologies has profoundly changed the way implant dentistry is planned, executed, and monitored [4, 5].

The introduction of three‐dimensional (3D) radiographic imaging, first through computed tomography (CT) and subsequently cone beam computed tomography (CBCT), marked a disruptive turning point in diagnostics. For the first time, clinicians were able to visualize the alveolar bone in three dimensions, assess anatomical risk structures with precision, and plan implant placement virtually rather than relying on two‐dimensional radiographs and tactile judgment. This innovation not only improved safety and predictability but also set the stage for the integration of digital workflows across the entire treatment process [6].

Building upon 3D imaging, computer‐assisted implant surgery (CAIS) emerged in two principal forms: static guidance (sCAIS), where surgical templates are produced based on virtual planning, and dynamic navigation (dCAIS), which enables real‐time tracking of implant drills during surgery [7, 8]. These technologies, though initially limited by cost, learning curves, and technical reliability, have steadily matured and are now increasingly used in daily practice. Their influence on precision and minimally invasive treatment strategies has been particularly notable in complex cases and full‐arch rehabilitation [9, 10].

The subsequent rise of intraoral scanning (IOS) further accelerated the digitalization of implant dentistry. By replacing conventional impressions, IOS has offered improved patient comfort, reduced clinical steps, and opened the possibility of fully digital workflows [11, 12, 13, 14]. Together with computer‐aided design and manufacturing (CAD/CAM) systems, IOS has revolutionized the way prosthetic reconstructions are designed and fabricated [15, 16, 17]. Milling technologies and, more recently, additive manufacturing (3D‐printing) have enabled a shift toward single‐visit dentistry and personalized solutions at an unprecedented level of precision [18, 19, 20, 21, 22]. Although single‐visit workflows are currently more established for subtractive manufacturing techniques, recent advances in additive manufacturing, including reduced printing and post‐processing times, have also enabled single‐visit dentistry for selected indications utilizing additive manufacturing techniques.

Today, digital implant dentistry encompasses all steps of the clinical workflow: from data acquisition, treatment planning, and guided or robotic surgery to digital impressions, CAD/CAM prosthetics, and maintenance [23]. The integration of these technologies has enhanced communication among clinicians, technicians, and patients, thereby improving treatment outcomes and patient satisfaction. Yet, this progress has also raised critical questions. How much digitalization does implant therapy truly need? Are high levels of technological investment justified in terms of patient benefit, cost‐efficiency, and accessibility? And what ethical considerations emerge as artificial intelligence (AI), robotics, and cloud‐based data systems increasingly shape clinical decision‐making?

This narrative review examines the evolution of digital implant dentistry across the entire clinical workflow, from diagnostics, planning, surgery and prosthetic restoration, up to patient monitoring, comparing historical and contemporary approaches at each treatment stage. To illustrate current clinical and technical protocols, three representative cases—single‐tooth replacement, partial edentulism, and complete edentulism—are presented step by step, demonstrating the application of digital technologies in modern implant therapy. Particular emphasis is placed on distinguishing truly disruptive innovations from more gradual technological advances, while also critically discussing emerging developments.

## 2 Historical Timeline

The evolution of digital implant dentistry is best understood as a sequence of overlapping technological waves rather than as a series of abrupt breaks. Each wave built on preceding discoveries—biological, mechanical, and digital—and together they gradually transformed implant therapy from an empirical, surgeon‐centered craft into an interdisciplinary, data‐driven specialty. Below key milestones are summarized in roughly decadal groupings to provide a compact timeline that frames the clinical workflow discussed in the remainder of this review.

The conceptual foundation was laid in the mid‐20th century with Per‐Ingvar Brånemark's experimental work on osseointegration and its clinical translation [1]. Over ensuing decades this biologic principle became the bedrock of reliable implant therapy and catalyzed broad acceptance of dental implants in routine practice [24]. In the decades that followed, established surgical techniques and conventional prosthetic workflows dominated clinical routines, supported primarily by two‐dimensional radiography and analogue laboratory processes.

The 1990s saw the first major digital diagnostic inflection point: 3D radiographic imaging entered clinical dentistry. Early medical CT provided three‐dimensional anatomical insight, and the subsequent development and dental optimization of CBCT in the late 1990s and early 2000s made volumetric imaging accessible, affordable, and practical for routine implant diagnostics [6]. This step permitted true three‐dimensional assessment of bone volume, angulation, and proximity to vital structures, and it constituted the technical prerequisite for virtual planning [25, 26, 27, 28].

Building on volumetric imaging, the 2000s introduced virtual implant treatment planning and the first clinically applied CAIS systems [29]. Static guided approaches (sCAIS), in which a surgical guide is fabricated from virtual plans, became established for increasing placement accuracy; dynamic navigation systems (dCAIS), which provide real‐time tracking of drills relative to the virtual plan, also emerged [30]. Concurrently, the early generations of intraoral scanners and CAD/CAM systems began to replace analogue impressions and manual lab work, enabling digital design of suprastructures and the milling of restorations with rising precision [12, 31, 32]. However, the broader clinical adoption of IOS in implant dentistry occurred at a later period after significant improvements of the systems.

The 2010s were characterized by maturation and convergence [33, 34, 35]. Substantial improvements in intraoral scanning accuracy, usability, and speed facilitated wider clinical adoption. CAD/CAM workflows expanded from chairside single‐unit restorations to laboratory‐driven digital prosthetics for implants. Also, additive manufacturing (3D printing) emerged as a practical method for producing surgical guides and as a promising, however not yet established, technology for fabricating provisional restorations and custom prosthetic components [4, 15, 19, 36, 37, 38, 39, 40]. Although additive manufacturing technologies started emerging in these years, they are nowadays still more often used for provisional restorations than definitive ones, due to the lack of a strong scientific evidence base. There is also a lack of comparative studies for printed and milled definitive restorations [18, 19]. Dynamic navigation systems improved in reliability, and early commercial robotic aids for implant placement appeared in prototype and regulated forms. In parallel, academic and industry research into automated image segmentation, AI‐assisted diagnostics, and automated design workflows began to produce viable tools for preclinical and some clinical tasks [41].

The early 2020s have continued these trends toward integration and automation [18]. Cloud‐based planning platforms, streamlined lab–clinic digital interfaces, and increasingly sophisticated software for implant planning and prosthesis design enable near‐seamless end‐to‐end digital workflows. AI has moved from proof‐of‐concept studies into practical assistance—supporting segmentation, landmark identification, and risk stratification [42, 43]. Advanced navigation systems are already used increasingly to translate virtual plans into more reproducible surgical execution. In this context, robotic systems were developed; however, their wide implementation in regular clinical practice is not achieved yet [44, 45, 46, 47, 48]. At the same time, innovations in sensors, intraoral monitoring, and telemetric data capture are promising to extend digital care into longitudinal maintenance and remote follow‐up [49, 50].

Taken together, this timeline illustrates a steady progression from biologic discovery (osseointegration) through diagnostic 3D imaging to contemporary, integrated digital workflows that span diagnosis, planning, execution, and maintenance. The pace of adoption has varied by geographical region, practice type, and economic considerations, but the technological trajectory is clear: incremental innovations have coalesced into a digitally enabled standard of care for many settings, even as work continues to determine which technologies are most disruptive, cost‐effective, and broadly beneficial in routine implant practice.

## 3 The Digital Implant Workflow

The digital implant workflow (Figure 1) was illustrated using three different clinical scenarios: a single‐tooth gap (Figures 2, 3, 4, 5, 6, 7), a partially edentulous patient (Figures 8, 9, 10, 11, 12), and a fully edentulous patient (Figures 13, 14, 15, 16, 17, 18). They only serve as illustrative examples rather than a comprehensive approach to all digital approaches.

**FIGURE 1:**
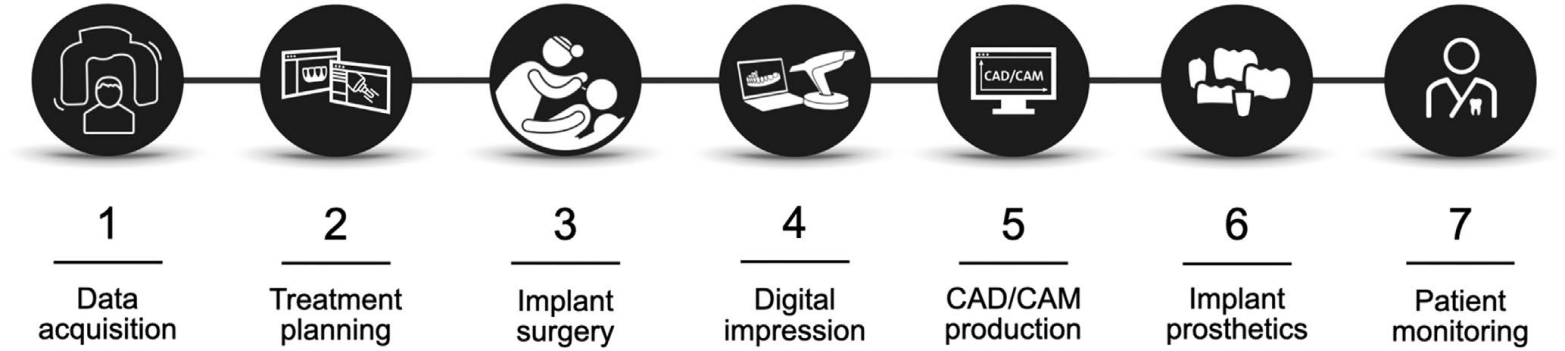
Digital implant workflow.

**FIGURE 2:**
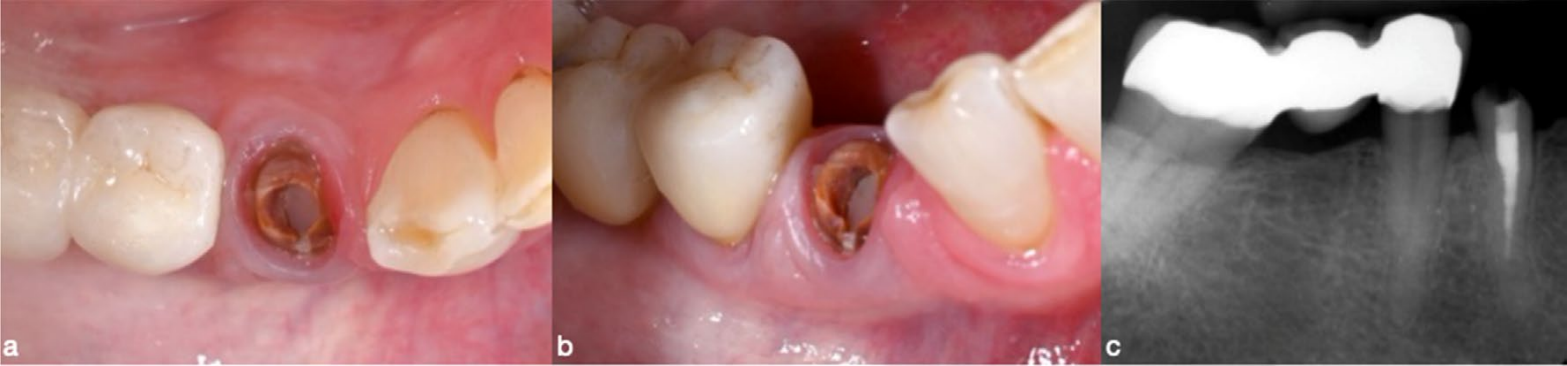
Initial patient data showing the fractured tooth 44 (a, b) occlusal/buccal aspects of the clinical situation, (c) initial periapical radiograph.

**FIGURE 3:**
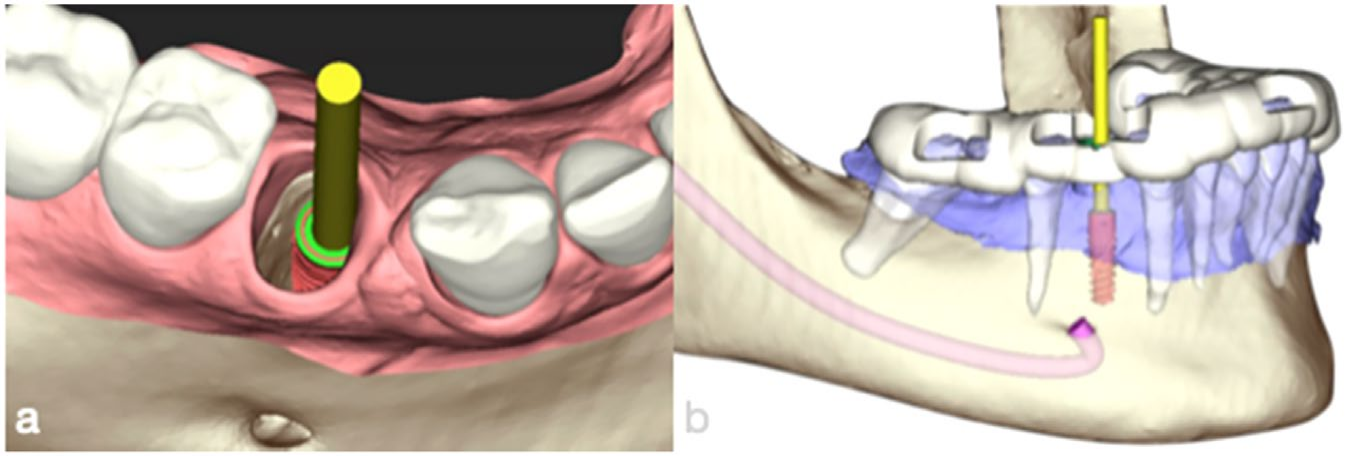
Virtual planning (coDiagnostiX; Straumann AG), for an immediate implant placement region 44 using sCAIS based on 3D data acquisition utilizing AI‐supported simulation of tooth extraction (a) superimposed CBCT/STL and prosthetically oriented backward planning, (b) 3D visualization of the final implant planning including surgical guide design.

**FIGURE 4:**
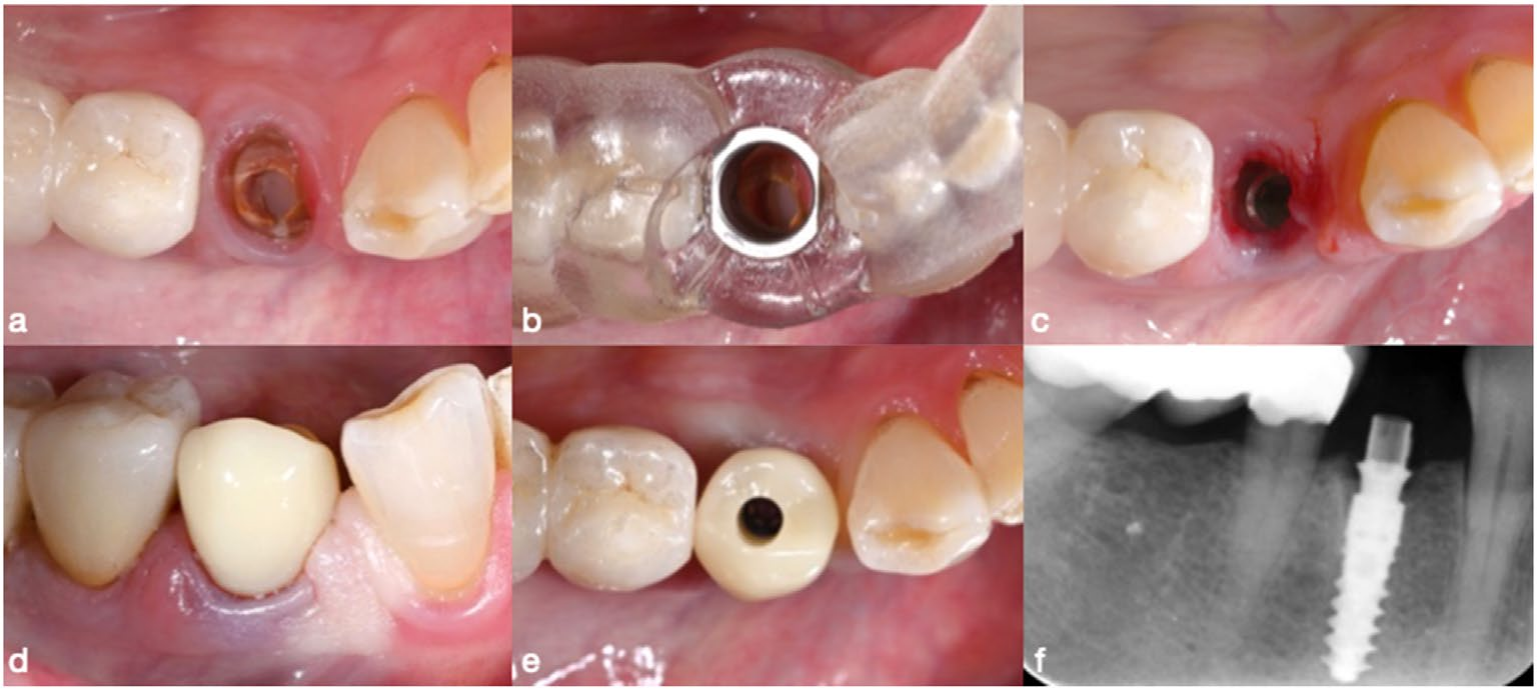
Surgical intervention for the immediate implant placement region 44 using sCAIS and a flapless approach (a) preoperative situation, (b) surgical guide for sCAIS in situ, (c) minimally traumatic tooth extraction and fully‐guided implant placement of a bone level implant (BLX; Straumann AG, Switzerland), (d, e) buccal/occlusal aspects with the milled screw‐retained immediate provisional implant‐supported prosthesis on a temporary abutment (RB/WB Temporary Abutment for crown; Straumann AG) with a gingival height of 2.5 mm in situ, (f) postoperative periapical radiograph.

**FIGURE 5:**
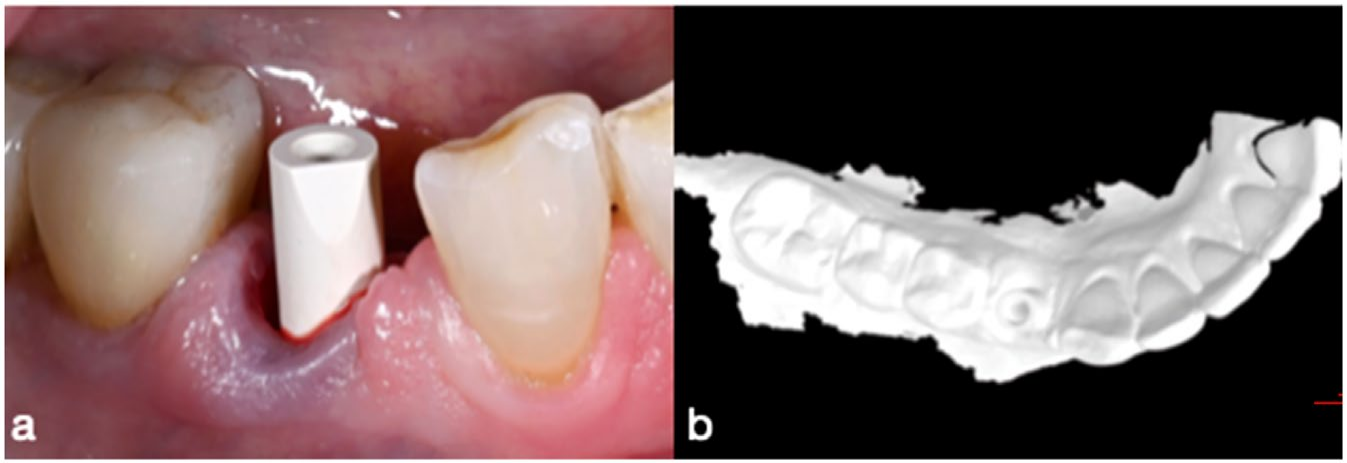
Intraoral data acquisition for prosthetic phase (a) clinical situation with scanbody (RB/WB Scanbody; Straumann AG) 44 in situ for digital impression, (b) STL‐file taken with intraoral scanning (TRIOS, 3Shape).

**FIGURE 6:**
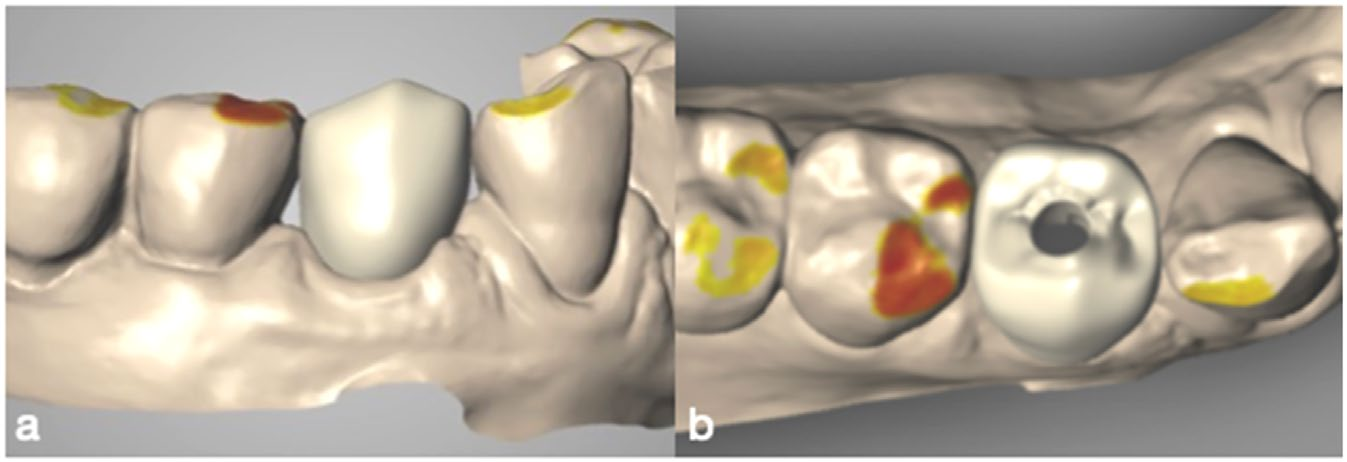
Computer‐aided design of the implant‐supported prothesis 44 (a) buccal aspect, (b) occlusal aspect.

**FIGURE 7:**
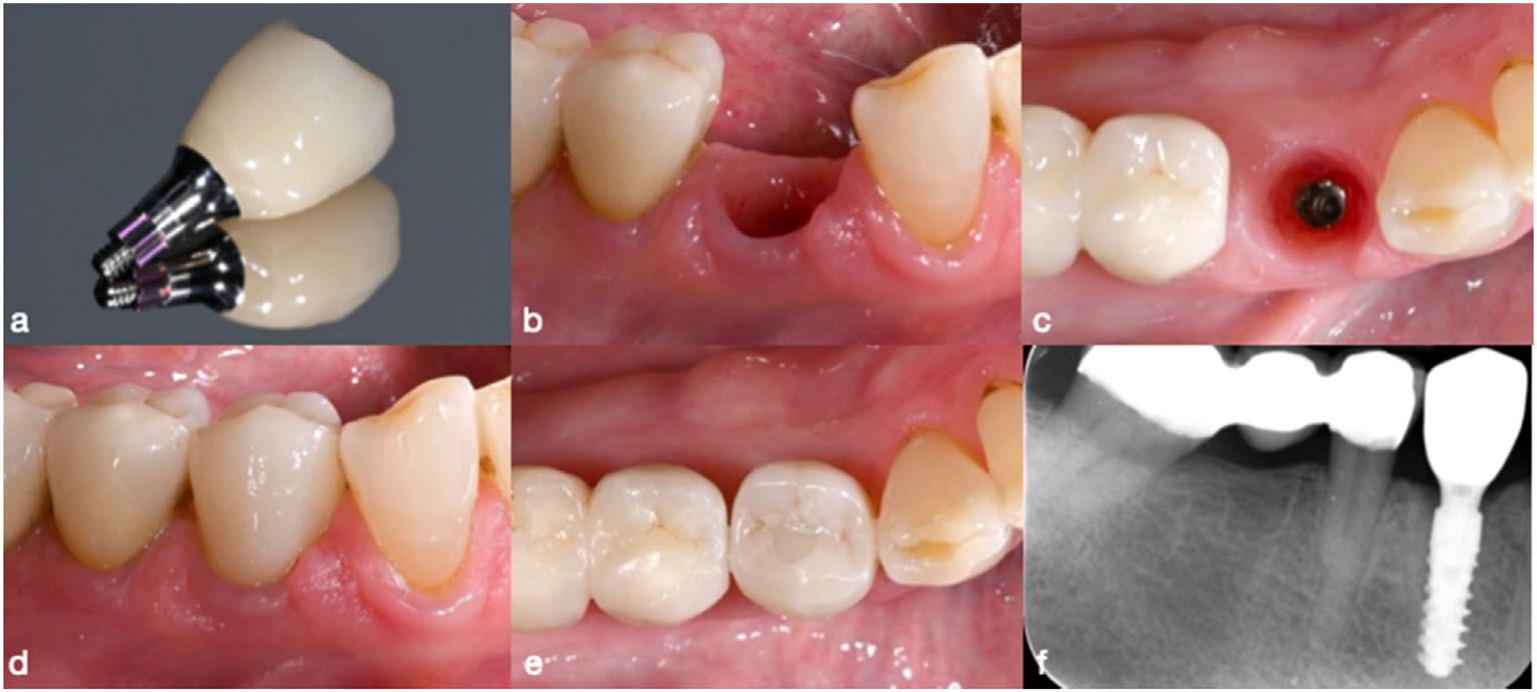
Delivery of the final implant‐supported restoration (a) milled screw‐retained monolithic implant‐supported single crown on a prefabricated titanium base abutment (RB/WB Variobase for Crown; Straumann AG) with a gingival height of 2.5 mm, (b, c) buccal/occlusal aspects showing soft‐tissue condition and emergence profile 6 weeks after immediate placement of implant 44, (d, e) buccal/occlusal aspects after delivery of the final restoration and composite filling of the screw‐access channel, (f) final periapical radiograph.

### 3.1 Data Acquisition

**Table:** 

Accurate diagnostic information is the cornerstone of successful implant therapy. The way in which clinicians acquire and interpret patient data has changed profoundly over the past three decades, evolving from two‐dimensional radiography to sophisticated three‐dimensional imaging and surface scanning technologies. This shift has fundamentally influenced treatment safety, predictability, and patient communication.

The past	The present
For much of the 20th century, diagnostic protocols in implant dentistry relied on 2D radiographs, as panoramic imaging and periapical films. These modalities could provide only limited information on bone volume and morphology, with distortions and superimpositions being common limitations. Treatment planning was often empirical, based on surgical experience and clinical judgment rather than objective volumetric data. Anatomical risks such as the mandibular canal, maxillary sinus, or the nasopalatine bundle could be estimated only indirectly, with higher risk resulting in surgical complications or compromised implant positioning. Supplementary techniques such as conventional tomography offered sectional images but were cumbersome associated with high‐dose radiation and rarely used in routine practice	The introduction of CBCT has transformed diagnostic protocols. Compared with medical CT, CBCT offers lower radiation exposure and compact devices suitable for dental offices. Modern CBCT scanners provide isotropic voxel sizes as small as 0.1 mm, enabling accurate assessment of bone dimensions, density, and proximity to anatomical structures. These datasets form the foundation for virtual implant planning software, in which implants can be positioned within a three‐dimensional model of the jaw. Increasingly, CBCT scans are combined with IOS to create merged datasets that represent both hard‐ and soft‐tissue surfaces with high fidelity. In this context, IOS has evolved into a versatile diagnostic tool.
From 2D radiographs and tactile estimation to CBCT and digital surface scanning, 3D data acquisition has fundamentally redefined implant diagnostics. What was once approximate has become measurable, and what was once empirical has become virtually reproducible. The ability to visualize patient‐specific anatomy in three dimensions does not merely support safer and more predictable surgery; it also enables a new form of patient communication, where virtual models make treatment options tangible and comprehensible. This transformation represents the first and perhaps most decisive step toward fully digital implant workflows.	

### 3.2 Treatment Planning

**Table:** 

Treatment planning in implant dentistry has always aimed to reconcile anatomical limitations with prosthetic requirements, but the tools and methods used to achieve this balance have changed profoundly over time. The evolution from two‐dimensional assessment and wax‐ups to fully digital, prosthetically driven planning exemplifies one of the most significant paradigm shifts in modern implantology.

The past	The present
Traditionally, implant planning was based on panoramic radiographs, supplemented by periapical films and study casts. Bone availability was estimated by measuring vertical height and horizontal width on distorted radiographs, often using radiographic markers or reference objects.	Today, treatment planning is largely digital and prosthetically driven. CBCT datasets can be merged with intraoral or laboratory scans, allowing precise alignment of bone anatomy with the intended tooth setups The tooth set‐up information can hereby be derived from a digitized wax‐up or a digital tooth arrangement. Specialized planning software enables clinicians to position implants in three dimensions relative to both anatomical structures and the intended prosthetic restoration.
The prosthetic perspective was incorporated by diagnostic wax‐ups or radiopaque stents, which helped visualize intended tooth positions on radiographs. However, this approach was inherently limited: measurements were imprecise, angulation was approximated, and discrepancies between radiographs and physical casts could lead to suboptimal implant positioning. In the posterior mandible, for example, uncertainty regarding the location of the inferior alveolar nerve often forced clinicians to accept shorter implants or wider safety margins, sometimes compromising restorative outcomes	This allows for optimized angulation, depth, and distribution of implants, thereby facilitating screw‐retained restorations and minimizing the need for customized abutments. The abutment selection can be performed during the digital planning as well. With studies showing the impact of abutment geometry and design on peri‐implant tissue stability [51, 52], this is a crucial step to consider during the treatment planning phase. Digital smile design and facial scans add a further dimension, particularly in the esthetic zone, by integrating soft‐tissue contours and facial proportions into the planning process [53]. These technologies present supportive approaches for a comprehensive treatment planning, however, these technologies do not necessarily essentially influence the treatment approach. The information from a digital smile design could also be created using conventional approaches and then digitalized to incorporate it into the digital planning. However, the use of a digital smile design significantly shortens laboratory fabrication time and allows for a mock‐up in situation where a conventional mock‐up try in is not possible. Nevertheless, digital smile design must be applied with caution, as simulated outcomes may not always be biologically or clinically achievable. In such cases, careful patient communication and expectation management are essential to avoid compromising patient satisfaction with the final result. The incorporation of a facial scan does not provide a significant or soundly proven benefit for the treatment planning, since also photographic documentation can provide sufficient information. Furthermore, facial scan data may be more difficult for patients to interpret and critically evaluate compared to conventional two‐dimensional images Collaboration between clinician and dental technician has also been transformed. Digital platforms allow real‐time communication and co‐planning, even across distances. Once finalized, the digital plan can be used directly to produce static surgical guides or exported into dynamic navigation systems, ensuring continuity between the virtual design and surgical execution. In addition, emerging artificial intelligence tools are beginning to support planning by automating steps such as tooth setup, nerve canal segmentation, or risk assessment, thereby reducing manual workload
Treatment planning has progressed from empirical approximations based on two‐dimensional images to comprehensive, prosthetically driven virtual workflows. This transformation has not only improved accuracy and safety but has also redefined the collaborative relationship between dentist, technician, and patient. In today's digital environment, planning is no longer a static preparatory step but an interactive and communicative process that bridges diagnostics, surgery, and prosthetics. Looking forward, the integration of artificial intelligence and automation is likely to further streamline and democratize this process, yet the clinician's judgment will remain central to ensuring that virtual plans translate into biologically and esthetically successful outcomes.	

### 3.3 Implant Surgery

**Table:** 

Surgical execution represents the pivotal moment where diagnostic data and virtual planning must be translated into a precise clinical outcome. Over the past decades, implant surgery has evolved from a predominantly freehand procedure guided by tactile experience into a digitally assisted process supported by static guides, dynamic navigation, and most recently robotic systems. Each stage of this evolution reflects the broader trend toward reproducibility, accuracy, and risk reduction.

The past	The present
In the early era of osseointegration, implant placement was performed entirely freehand. Clinicians relied on panoramic radiographs, periodontal probes, and surgical templates with rudimentary radiopaque markers to approximate the intended position. Surgical access was often achieved via large mucoperiosteal flaps, permitting direct visualization of bone structures. Although this approach allowed flexibility, its precision was highly dependent on the operator's skill and experience. Marginal errors in angulation or depth could lead to restorative challenges, particularly in the esthetic zone, where mispositioned implants compromised soft‐tissue harmony. In posterior sites, inadequate visualization of the mandibular canal or maxillary sinus occasionally resulted in complications	The introduction of computer‐assisted implant surgery (CAIS) has fundamentally reshaped the surgical landscape. In static CAIS (sCAIS), CBCT and intraoral scan data are combined in planning software to design a surgical template, which is then fabricated via CAD/CAM milling or 3D printing These guides allow the transfer of virtual implant positions into the patient with high accuracy, especially in straightforward cases such as single posterior implants. They might require additional stabilization with fixation pins in fully edentulous patients. In dynamic CAIS (dCAIS), optical or electromagnetic tracking systems provide real‐time visualization of the drill in relation to the patient's anatomy and the virtual plan. This method allows intraoperative flexibility while maintaining accuracy, making it particularly valuable in anatomically demanding or full‐arch cases. Overall, static navigation systems appear to be more widely implemented and used than dynamic ones. Recent years have also seen the emergence of robotic‐assisted systems, which can stabilize drills, restrict motion to pre‐planned trajectories, execute osteotomies semi‐automatically under clinician supervision or even perform the implant placement itself. Although still limited in availability and cost‐intensive, these technologies represent a potential future paradigm for standardized, minimally invasive implant placement. However, they still require further scientific evaluation. Furthermore, in cases requiring flap elevation and simultaneous bone grafting, human surgical intervention remains necessary, as the robotic systems are currently limited to performing osteotomy and implant placement.
Implant surgery has progressed from freehand placement guided by clinical intuition to digitally assisted workflows that aim to replicate virtual plans with millimetric accuracy. Although CAIS and robotics expand the surgical armamentarium, their value lies not in replacing surgical judgment but in enhancing it. The clinician's ability to integrate digital precision with biological and prosthetic principles will continue to define the quality of implant care. Ultimately, digital surgery is not an end in itself but a means to deliver safer, more predictable, and more esthetically satisfying outcomes for patients.	

### 3.4 Digital Impression

**Table:** 

Impressions form the critical link between surgical execution and prosthetic rehabilitation. They capture the intraoral situation, transfer it to the laboratory, and thereby define the precision of the final restoration. Over the past decades, this step has undergone one of the most visible transformations in dentistry: from physical impression materials and stone casts to intraoral scanning and fully digital datasets.

The past	The present
For most of the 20th century, implant impressions were made using conventional impression materials such as polyether or polyvinyl siloxane, often combined with open‐ or closed‐tray transfer copings. Accuracy was generally high for single units, but multiple implants, divergent angulations, or full‐arch cases often introduced significant error due to impression material distortion, tray flexure, or inaccuracies during pouring of stone models. Conventional workflows also involved time‐consuming laboratory steps—pouring, trimming, mounting—each with the potential for cumulative inaccuracies. Patient comfort was another concern, as large trays and viscous impression materials were often unpleasant, particularly for edentulous patients	Digital impression techniques using intraoral scanners (IOS) have increasingly replaced conventional methods. Modern IOS systems capture high‐resolution optical data of teeth, soft tissues, and implant scan bodies, creating accurate digital models without the need for impression trays or materials. For single‐unit and short‐span implant cases, IOS has achieved accuracy comparable to, or even surpassing, conventional impressions. The workflow is streamlined: scan data can be transferred directly to the laboratory or CAD/CAM system, enabling faster turnaround and eliminating the risk of plaster or model errors. Long‐span edentulous areas or fully edentulous cases present a challenging indication for the use of a an IOS, since the accuracy might be limited in these cases.
	In long‐span or full‐arch cases the accuracy of the digital impression might be affected by the jaw location and the distances between the implants. For the master model fabrication after implant placement in long‐span or full‐arch cases it is recommended to reevaluate scan accuracy using verification jigs [54]. Photogrammetry presents an additional technology to capture the implant position in full‐arch cases. The addition of a photogrammetry scan be used to support the accuracy of a master model in full‐arch cases. However, it still requires an intraoral digital impression to capture the soft tissue anatomy. In addition to accuracy and efficiency, IOS offers new diagnostic and monitoring opportunities. Digital impressions can be stored longitudinally, allowing comparison of peri‐implant soft‐tissue changes over time. For patients, the comfort and immediacy of digital impressions enhance acceptance, while real‐time visualization supports communication and shared decision‐making.
The transition from analogue to digital impressions exemplifies the broader transformation of implant dentistry. What was once a laborious and material‐intensive process has become a fast, comfortable, and data‐rich procedure that seamlessly integrates with CAD/CAM production. Although certain challenges remain—particularly in complex full‐arch cases—the trajectory is clear: digital impressions are becoming the new standard, not only because of accuracy and efficiency but also because they enable longitudinal data collection and new dimensions of patient care. In this sense, digital impression technology is more than a replacement for conventional methods; it is a gateway to a fully digital, patient‐centered workflow.	

### 3.5 CAD/CAM Production

**Table:** 

Once digital impressions have been captured, the next crucial step is the transformation of this data into physical restorations. Here, computer‐aided design and computer‐aided manufacturing (CAD/CAM) technologies have profoundly altered how implant prosthetics are conceived, fabricated, and delivered. This transformation has extended beyond mere efficiency; it has reshaped material choices, accuracy, and the collaboration between clinic and laboratory.

The past	The present
Traditionally, implant prosthetics were produced through analogue laboratory processes. After stone casts were created from conventional impressions, wax‐ups were used to design the suprastructure. Metal frameworks were laboriously fabricated through casting, often requiring multiple try‐ins to ensure passive fit. Veneering with ceramics or composites was performed manually, making outcomes highly dependent on technician skill. Although excellent restorations could be produced, the process was time‐intensive, costly, and prone to inaccuracies due to cumulative errors in impression, casting, and model handling	CAD/CAM has streamlined and standardized the production of implant‐supported restorations. Once digital data from intraoral scanning is available, prosthetic design is carried out virtually, with software offering precise control of emergence profiles, occlusion, and screw‐channel positioning. The finalized design can be manufactured via subtractive milling or additive 3D printing, depending on material and indication. For single‐tooth and short‐span restorations, chairside CAD/CAM systems allow same‐day delivery, reducing treatment time and enhancing patient satisfaction. In more complex cases, centralized laboratories or milling centers can produce highly accurate frameworks in titanium, cobalt‐chromium, or zirconia. Additive manufacturing has further expanded possibilities, enabling the fabrication of surgical guides, custom abutments, and provisional prosthetics with great flexibility. Although subtractively manufactured restorations are well documented, scientific documentation for the long‐term clinical performance of 3D printed definitive restorations is currently lacking. Therefore, 3D printed restorations are at the moment rather used for provisional restorations than definitive restorations. Importantly, CAD/CAM production facilitates a level of standardization and reproducibility that was difficult to achieve with purely manual workflows.
CAD/CAM has transformed implant prosthetics from artisanal craftsmanship into digitally guided precision manufacturing. By merging digital design with industrial‐level fabrication technologies, restorations are now produced more accurately, reproducibly, and efficiently than ever before. Yet, the success of CAD/CAM does not lie solely in its ability to automate; it also lies in its integration of clinician, technician, and patient into a continuous digital chain. In this sense, CAD/CAM production is both a technological advance and a cultural shift—one that has elevated implant prosthetics into the realm of high‐precision, digitally enabled healthcare.	

### 3.6 Implant Prosthetics

**Table:** 

The prosthetic phase represents the culmination of the digital workflow: translating diagnostic data, surgical precision, and CAD/CAM production into a functional and esthetic restoration. In many ways, the quality of the prosthetic outcome determines the overall success of implant therapy, since it directly affects patient comfort, appearance, and long‐term function.

The past	The present
In the analogue era, prosthetic workflows for implants were complex and heavily technique‐sensitive. Frameworks were cast in metal, veneered with ceramics or composites, and manually adjusted to achieve occlusal balance and esthetic integration. Screw‐retained restorations were often avoided in favor of cement‐retained designs, since the precision required to align screw channels with occlusal surfaces was difficult to achieve freehand. Although excellent prosthetics could be achieved, this required extensive laboratory craftsmanship, multiple clinical appointments, and often significant chairside adjustments. Long‐term complications—such as peri‐implantitis linked to excess cement, or mechanical failures from misfit frameworks—were relatively common	Digital workflows have reshaped implant prosthetics. Virtual planning and CAD/CAM manufacturing enable restorations that are designed prosthetically from the outset, often favoring screw‐retained solutions with optimal channel positioning. Nowadays, majority of implant‐supported restorations utilize titanium‐base abutments. Digital libraries of these titanium‐base abutments allow their integration into the CAD/CAM process Alternatively, customized abutments can be digitally designed to support soft‐tissue contours and emergence profiles, particularly in the esthetic zone. Monolithic restorations in zirconia or lithium disilicate, produced via milling, offer strength, esthetic translucency, and precision. For an optimal esthetic integration, they might require a minimal layer of buccal veneering for characterization in the esthetic zone. For full‐arch rehabilitations, milled titanium or zirconia frameworks provide superior passive fit and biomechanical stability compared to traditional cast frameworks. An additional advantage of digital prosthetics lies in reproducibility. Once a design is digitally archived, it can be modified, re‐milled, or 3D‐printed if repairs or replacements are required. This continuity is particularly valuable in full‐arch cases, where complications such as veneer fractures or screw loosening may necessitate intervention years after initial delivery.
Implant prosthetics have evolved from labor‐intensive analogue craftsmanship to digitally designed and manufactured solutions that are more accurate, reproducible, and biologically favorable. Although technology has reduced variability and improved outcomes, the clinician's role in harmonizing digital precision with biological and esthetic principles remains indispensable. In essence, digital prosthetics embody the convergence of science, technology, and artistry—transforming implant‐supported restorations from technical replacements into integrated, patient‐centered reconstructions.	

### 3.7 Patient Monitoring

**Table:** 

Implant therapy does not end with prosthetic delivery. Long‐term success depends on structured maintenance and monitoring, which safeguard peri‐implant health, identify complications early, and preserve both function and esthetics over decades. In this domain, the transition from analogue follow‐up protocols to digital monitoring represents one of the most promising—yet still evolving—dimensions of implantology.

The past	The present
Historically, maintenance relied on periodic clinical examinations combined with conventional two‐dimensional radiographs. Probing depth, bleeding on probing, and mobility tests were the primary tools to assess peri‐implant status. Stone casts or photographs provided occasional reference for prosthetic wear or soft‐tissue changes, but longitudinal comparison was often imprecise. Complications such as peri‐implant mucositis or early bone loss were frequently diagnosed only once clinically evident, limiting the possibility of preventive intervention Follow‐up intervals and documentation were highly variable, depending more on the clinician's preference than on standardized protocols	Digital technology has broadened the scope of maintenance. Intraoral scanning enables highly precise, non‐invasive documentation of peri‐implant soft tissues and prosthetic surfaces over time. By superimposing scans from different recall appointments, clinicians can quantify changes in gingival contours, recession, or wear patterns with submillimeter accuracy. Radiographic follow‐up has also benefited from digitalization, with standardized CBCT or digital periapicals offering reproducible reference images. Although not widely implemented yet, artificial intelligence tools are beginning to support this process by automating the detection of bone‐level changes or identifying risk factors such as residual cement or excessive prosthetic contours. In addition, digital platforms facilitate structured data storage, enabling both longitudinal follow‐up of individual patients and large‐scale outcome analysis across clinical populations.
Maintenance and monitoring are entering a digital era in which longitudinal precision, reproducibility, and predictive analytics increasingly complement traditional clinical parameters. The shift from subjective observation to data‐rich documentation enables earlier intervention, improved patient communication, and more personalized care. At the same time, these tools highlight that implant therapy is not a finite event but a continuum—one in which digital technologies may transform aftercare into an active, predictive, and patient‐specific process.	

The relevance of digital workflows may vary depending on the clinical complexity and treatment objectives. In straightforward single‐unit posterior cases (Figures 2, 3, 4, 5, 6, 7), implant placement can be achieved predictably using either a digital guided or a conventional freehand approach. In the present case, however, the digital workflow facilitated the fabrication and delivery of an immediate provisional restoration. In more complex clinical situations, such as multiple‐unit rehabilitations or treatments in esthetically demanding areas (Figures 8, 9, 10, 11, 12, 13, 14, 15, 16, 17, 18), digital planning and guided surgery may offer additional advantages by supporting prosthetically driven implant positioning and enhancing treatment planning and execution. Furthermore, CAD/CAM‐based restorative workflows contributed to the efficient fabrication of the prosthetic components used in the presented cases.

**FIGURE 8:**
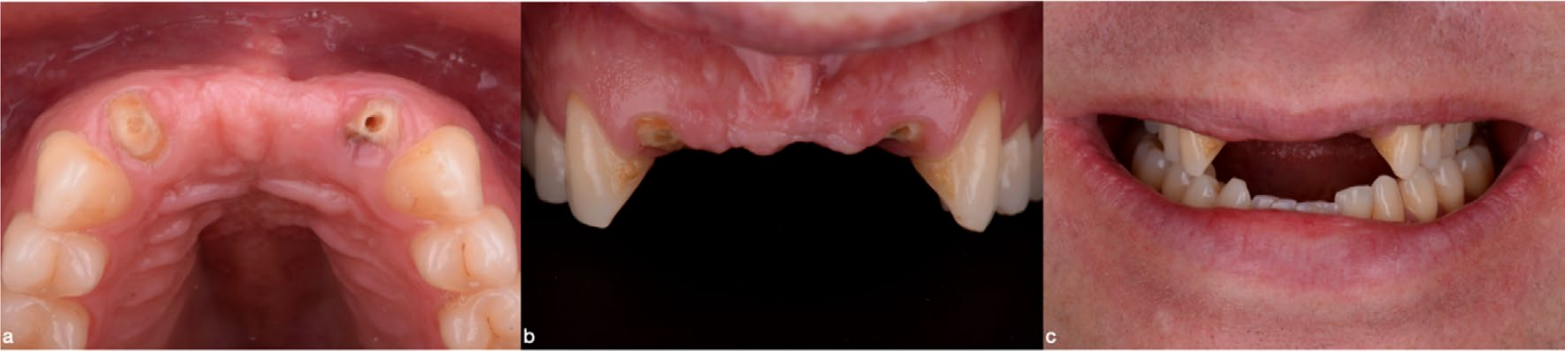
Initial patient data showing the fractured teeth 12 and 22 (a, b) occlusal/buccal aspects of the clinical situation, (c) extra‐oral photograph.

**FIGURE 9:**
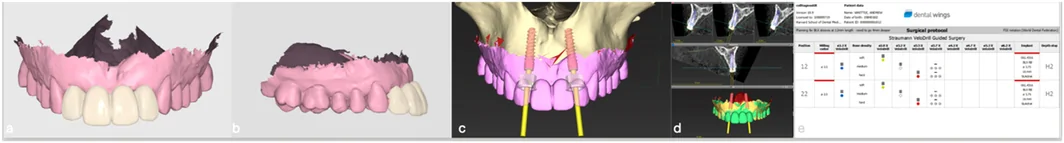
Virtual planning based on 3D data acquisition (a, b) digital tooth arrangement, (c, d) superimposed CBCT, STL, digital tooth arrangement, and implant planning using an implant planning software (coDiagnostiX; Straumann AG), (e) drill protocol.

**FIGURE 10:**
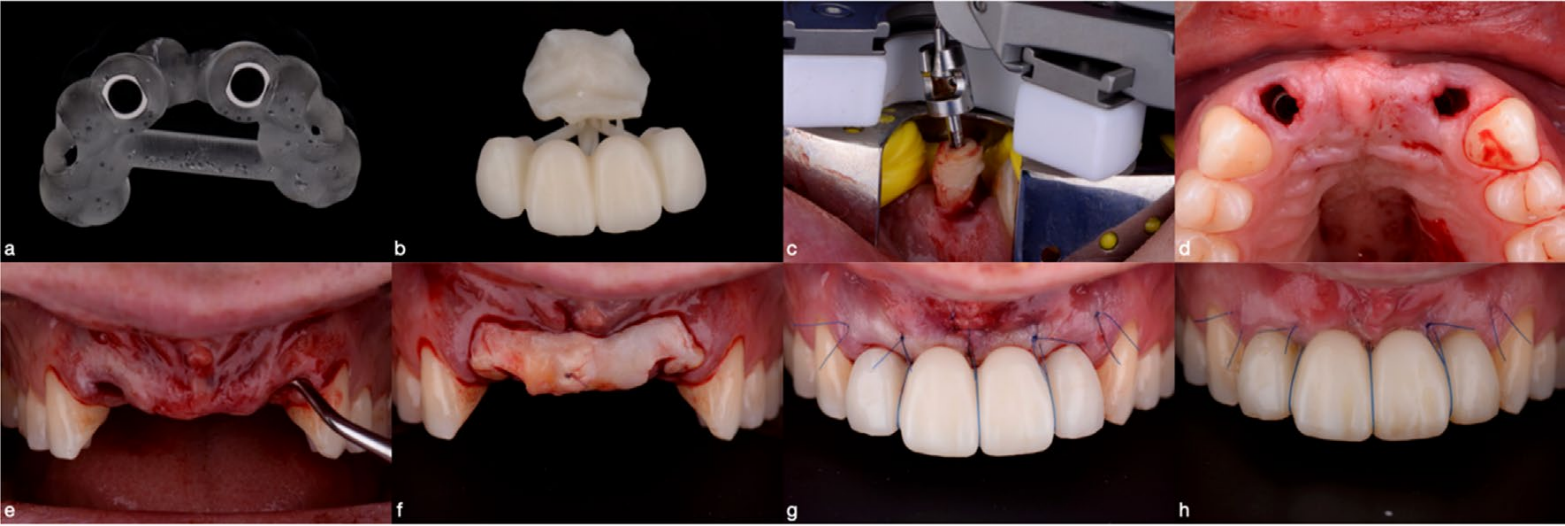
Surgical intervention (a) surgical guide for s‐CAIS, (b) provisional restoration for an immediate loading procedure, (c) tooth extraction utilizing a vertical tooth extraction device for a minimally traumatic tooth extraction, (d) guided implant placement of 2 bone level implants (BLX; Straumann AG) region 12 and 22, (e) tunneling, (f) soft tissue augmentation procedure, (g) suturing and immediate loading of implants with a screw‐retained iFDP, (h) follow‐up 2 weeks after surgical intervention.

**FIGURE 11:**
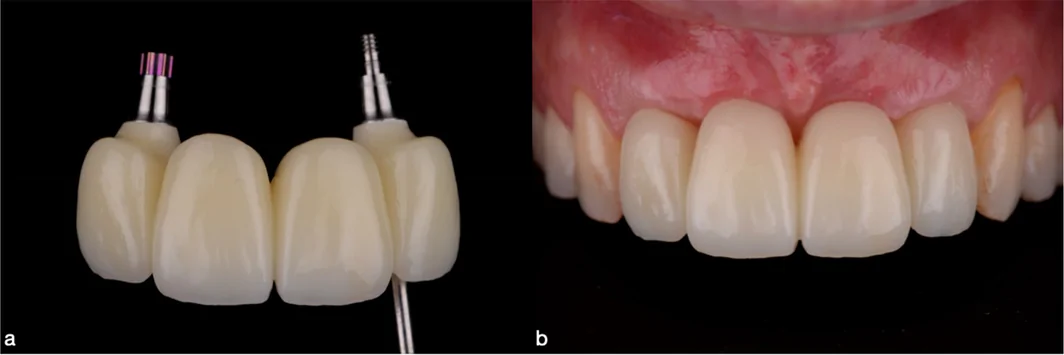
(a) Milled screw‐retained provisional implant‐supported prosthesis on prefabricated titanium base abutments (RB/WB Variobase; Straumann AG), (b) clinical situation with provisional implant‐supported prosthesis in situ.

**FIGURE 12:**
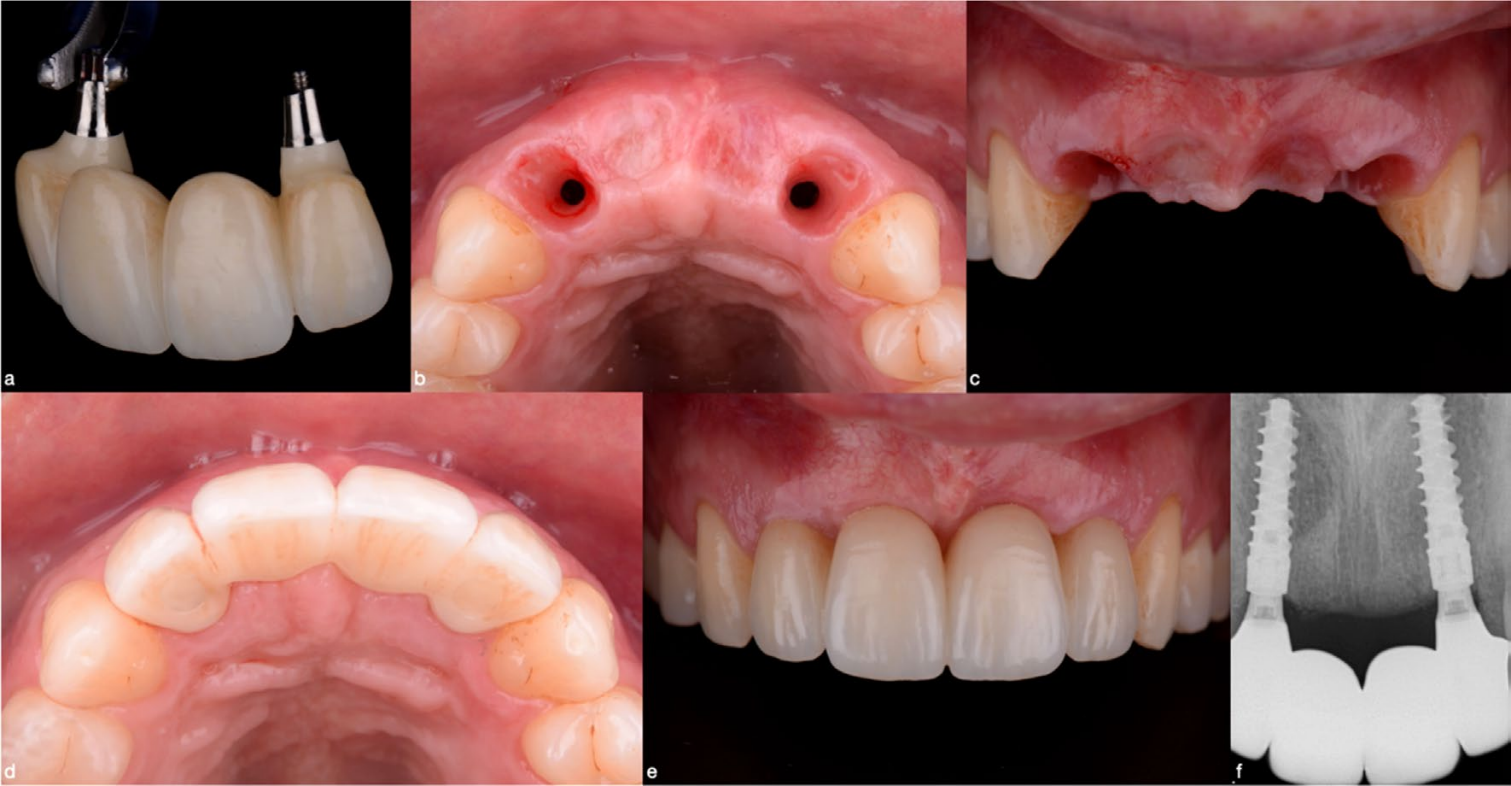
Delivery of the implant‐supported prosthesis (a) milled screw‐retained implant‐supported prosthesis on prefabricated titanium base abutments (RB/WB Variobase; Straumann AG), (b, c) occlusal/buccal aspects of the clinical situation after soft tissue conditioning, (d, e) occlusal/buccal aspects of the clinical situation after delivery of the implant‐supported prosthesis, (f) final radiograph.

## 4 The Future of Implant Dentistry

The future of implant dentistry will be shaped by digital technologies that extend beyond the current clinical workflow and open entirely new dimensions of diagnosis, planning, and patient care [55]. Among the most transformative developments is the integration of artificial intelligence into diagnostics and treatment planning. Algorithms capable of analyzing large datasets of radiographs, intraoral scans, and clinical outcomes are beginning to support clinicians in identifying risk factors, predicting treatment success, and proposing prosthetically driven implant positions with remarkable precision. Although such systems are still in their infancy, they show rapid development and are expected to be increasingly used in implant dentistry in the near future. Their potential lies in transforming complex diagnostic reasoning into accessible, data‐driven decision support, thereby reducing variability and enhancing treatment predictability [56].

Equally disruptive is the advent of robotics in implant surgery. Dynamic navigation has already paved the way toward real‐time, computer‐assisted guidance, but robotic platforms are poised to take precision one step further by offering haptic feedback, motion stabilization, and semi‐autonomous drilling protocols [57]. These systems may reduce human error, improve safety in anatomically demanding cases, and potentially lower the learning curve for less experienced surgeons. However, the integration of robotics also raises critical questions about workflow efficiency, cost, and the evolving role of the clinician in balancing human judgment with machine assistance. Although these systems might be in the appropriate developmental stage soon for broader clinical use, it is to be expected that it will still require a more extended period until they are implemented broadly, due to cost reasons and potential hesitation on the sides of the clinicians as well as the patients [58, 59].

Another frontier is the use of intraoral sensors for individualized long‐term monitoring [60]. Currently still in the developmental stage, such sensors, embedded within prosthetic components, could continuously track parameters such as occlusal forces, temperature fluctuations, or biochemical markers of inflammation in peri‐implant crevicular fluid. Currently, most reported sensors focus on bite force, pressure application, and temperature changes [60]. Biomarkers are currently predominantly used in periodontology for monitoring disease prevention and progression. They hold future potential if they can be implemented in precision dentistry and personalized workflows [59]. By alerting both patients and clinicians to early signs of overload or disease activity, sensor‐based monitoring could enable a paradigm shift from reactive treatment of complications to proactive maintenance [60]. This would not only enhance the longevity of implants but also empower patients to take an active role in their oral health through personalized digital feedback. Their broader use in dentistry is expected to require a longer period due to needed further proof‐of‐concept and development.

Perhaps the most visionary development is the integration of all digital records—radiographic datasets, surface scans, prosthetic designs, surgical parameters, and monitoring data—into a comprehensive “digital twin” of the patient [61]. Such a virtual replica would allow simulation of biological and mechanical responses before clinical interventions are undertaken, enabling predictive modeling of long‐term outcomes. Whereas radiographic datasets, surface scans, digital tooth arrangements, and surgical parameters are already being registered and used in implant dentistry, the addition of monitoring data is still in the developmental stage [62, 63, 64]. In the context of implant dentistry, this concept could mean simulating bone remodeling, prosthetic wear, or soft‐tissue dynamics under different treatment scenarios, thereby providing clinicians with a powerful tool for patient‐specific decision‐making and informed consent.

Taken together, these innovations signal a future in which digital implant dentistry is not limited to improving precision and efficiency in existing workflows, but rather evolves into a data‐driven, patient‐specific ecosystem. Artificial intelligence, robotics, sensor‐based monitoring, and the digital twin all represent building blocks of a new paradigm that extends beyond the replacement of missing teeth toward the holistic integration of oral rehabilitation into personalized healthcare.

## 5 Discussion

The digital transformation of implant dentistry has fundamentally reshaped the way clinicians diagnose, plan, and execute treatment [4]. Yet, as with every technological shift, the challenge lies not only in identifying the advantages but also in critically appraising which innovations have truly been disruptive, which have followed an evolutionary trajectory, and where the balance between digital and analogue methods should ultimately be drawn.

Few technologies can be regarded as genuinely disruptive in the sense that they redefined the standards of care. Cone‐beam computed tomography (CBCT) was arguably the first of these milestones [6]. Its introduction transformed implant diagnostics by enabling three‐dimensional visualization of bone anatomy, adjacent structures, and prosthetic planning, replacing the limitations of two‐dimensional radiography [6, 28]. A second disruptive leap was the arrival of intraoral scanning, which eliminated the need for conventional impressions, reduced patient discomfort, and introduced an entirely new way of documenting the dentition and soft tissues [11]. Closely linked to this development, CAD/CAM technology in prosthetic production revolutionized restorative dentistry by ensuring reproducibility, precision, and efficiency while opening the door to monolithic and additive workflows [12, 15, 19, 36, 39]. These innovations did not merely refine existing methods but altered clinical protocols so fundamentally that contemporary implantology can scarcely be imagined without them.

Other technologies, while valuable, can be considered more evolutionary. Virtual treatment planning software [3, 65], for example, expanded the possibilities introduced by CBCT, but in many cases still relies on the clinician's experience and judgment. Computer‐assisted implant surgery (CAIS), both static and dynamic, has certainly enhanced accuracy and safety, yet it often complements rather than replaces conventional freehand placement [8, 30]. Similarly, advances in digital prosthetic design and milling strategies, though continuously improving, represent iterative refinements rather than paradigm shifts. The distinction between disruptive and evolutionary innovations underscores the uneven pace of digitalization, with some domains transformed overnight and others advancing more gradually.

This raises the question of how much digitalization implant dentistry truly requires. There is no doubt that digital tools improve diagnostic accuracy, surgical precision, and restorative efficiency. Yet, the ultimate goal of implant therapy—restoring oral function, comfort, and esthetics—can still be achieved without fully digital workflows, provided the clinician is skilled and experienced. Indeed, in many parts of the world, implant therapy remains largely analogue, and good outcomes are achieved without the latest digital resources. What digitalization offers, however, is the ability to standardize quality, reduce variability, and expand access to high‐precision treatments beyond centers of excellence. In this sense, the digital shift is less about replacing analogue competence than about democratizing predictable outcomes.

Nevertheless, digitalization comes at a cost—not only financially but also in terms of time, training, and ethical responsibility. The acquisition of CBCT scanners, intraoral scanners, and CAD/CAM systems requires substantial investment, which may not be feasible for all practitioners, particularly in smaller practices or in regions with limited resources. Moreover, the learning curve associated with mastering digital workflows is steep and demands continuous education. There is a risk that the increasing technological complexity of implant dentistry may widen the gap between early adopters and those who cannot, or choose not to, invest in such systems.

Beyond economic barriers, ethical considerations emerge as digital data flows increasingly through cloud‐based platforms and AI‐supported environments [66]. Questions of data ownership, patient consent, and cybersecurity become integral to clinical decision‐making. Although digital records promise efficiency and long‐term monitoring, they also expose sensitive health information to risks of misuse or unauthorized access. Similarly, the integration of artificial intelligence into diagnostic and treatment planning raises questions about accountability: if an AI‐based recommendation influences clinical decisions, who bears responsibility for the outcome—the clinician, the software provider, or both? These issues underline the need for robust regulatory frameworks that safeguard both patients and practitioners [66, 67].

In sum, the digital transformation of implant dentistry is neither uniformly disruptive nor universally necessary. It is a complex evolution in which certain breakthroughs—CBCT, IOS, CAD/CAM—have irreversibly changed practice, while other innovations offer incremental benefits that must be weighed against costs and ethical implications. The central challenge for the profession lies in embracing digital tools as powerful enablers while avoiding the trap of technology for its own sake. Implant dentistry remains a discipline in which clinical expertise, patient‐centered care, and critical judgment are irreplaceable. Digitalization should therefore be seen not as an endpoint, but as an evolving toolbox—one that must be applied thoughtfully, selectively, and responsibly.

## 6 Conclusions

Digital implant dentistry has undergone a remarkable evolution, from the analogue beginnings of the Brånemark era to the precision of today's data‐driven workflows. The past was defined by pioneering efforts and 2D techniques, the present by the integration of CBCT, IOS, and CAD/CAM into daily practice, and the future by the promise of AI, robotics, and sensor‐based patient monitoring. What emerges is not a replacement of clinical expertise, but an expansion of the clinician's toolbox, empowering more predictable, patient‐centered, and personalized implant therapy (Figure 19).

- CBCT, IOS, and CAD/CAM represent the most disruptive milestones, transforming diagnostics, planning, surgical, and prosthetic protocols.
- Virtual planning software, computer‐assisted implant surgery, and advances in digital prosthetic design and milling strategies present evolutionary technologies.
- Digital innovations offer incremental benefits, but should be critically weighed against costs, learning curves, and clinical necessity.
- Implant therapy remains possible without digital tools, yet digitalization enhances standardization, safety, predictability, and patient experience.
- The future lies in integrating AI, robotics, and intraoral sensors into a holistic “digital twin” approach that supports personalized implant therapy.
- Ethical and economic considerations—data security, investment, and accessibility—will shape the pace of adoption.

**FIGURE 13:**
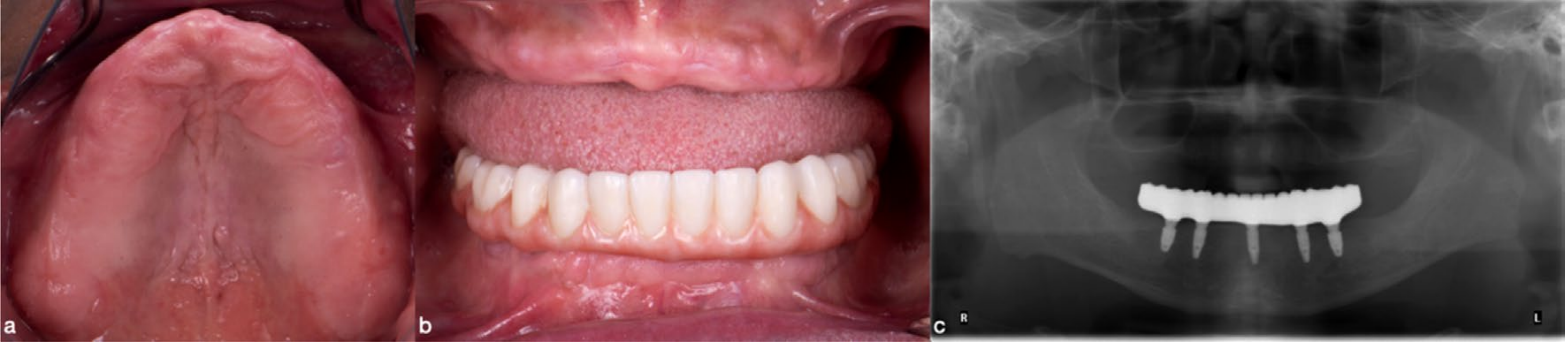
Initial patient data (a, b) occlusal/buccal aspects of the clinical situation, (c) initial radiograph.

**FIGURE 14:**
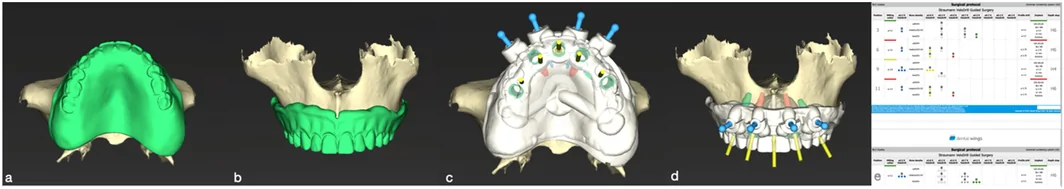
Virtual planning (coDiagnostiX; Straumann AG), based on 3D data acquisition (a, b) occlusal/buccal aspects of the digital tooth arrangement, (c, d) superimposed CBCT, STL, digital tooth arrangement, implant planning, and surgical guide, (e) drill protocol.

**FIGURE 15:**
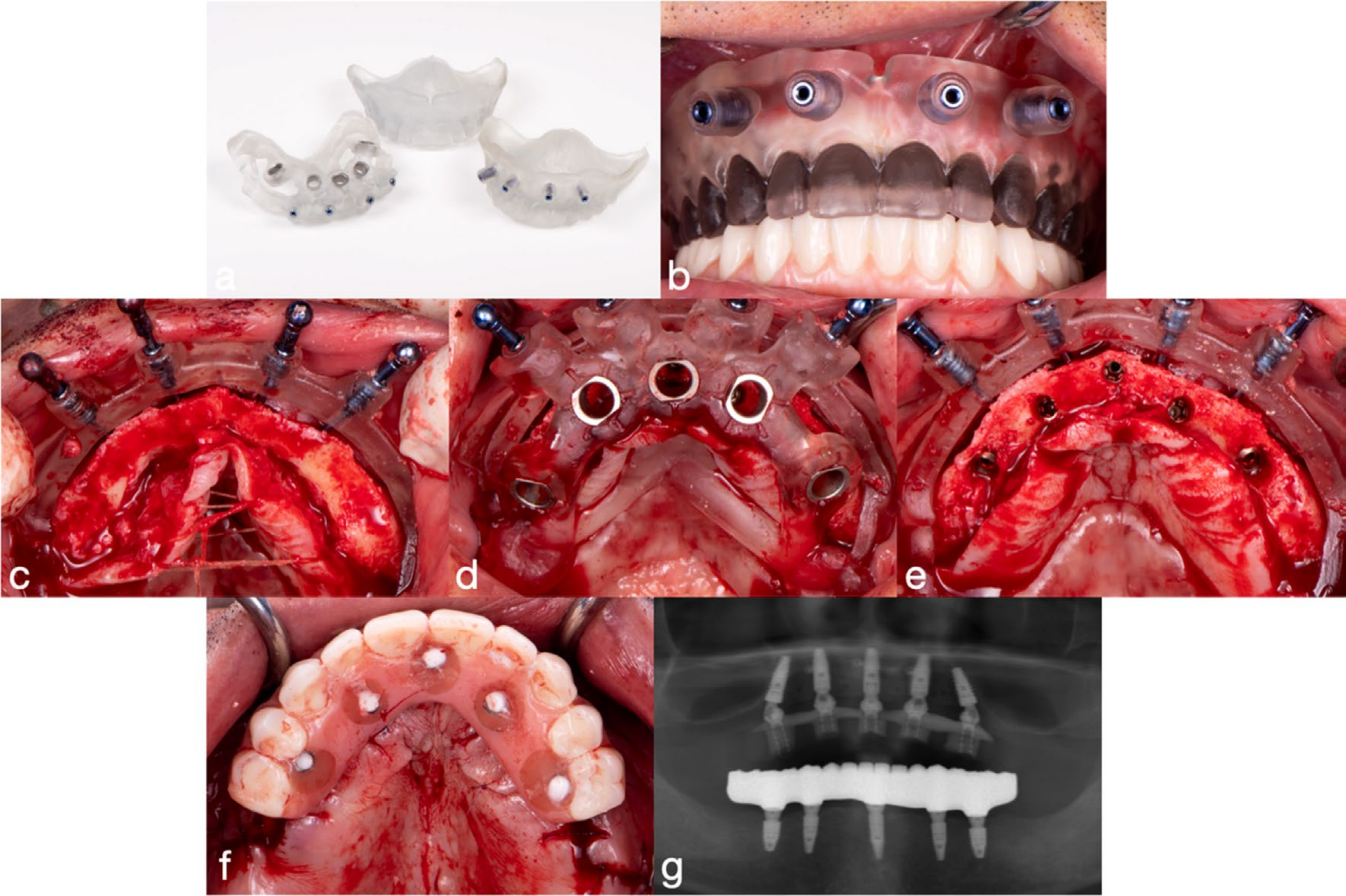
Surgical intervention (a) stackable surgical guide for s‐CAIS with a guide for the correct positioning of the fixation pins, the bone reduction and implant placement, (b) surgical guides in occlusion to allow correct positioning of the guide to place the fixation pins, (c) inserted fixation pins, flap elevation and bone reduction to create adequate prosthetic space, (d, e) implant placemen of 5 bone level implants (BLC; Straumann AG), (f) after insertion of the multi‐unit abutments (SRA straight and angled; Straumann AG), immediate loading of the implants with a provisional implant‐supported prosthesis on temporary abutments (Tan Coping; Straumann AG) and using an intra‐oral pick‐up approach, (g) postoperative radiograph.

**FIGURE 16:**
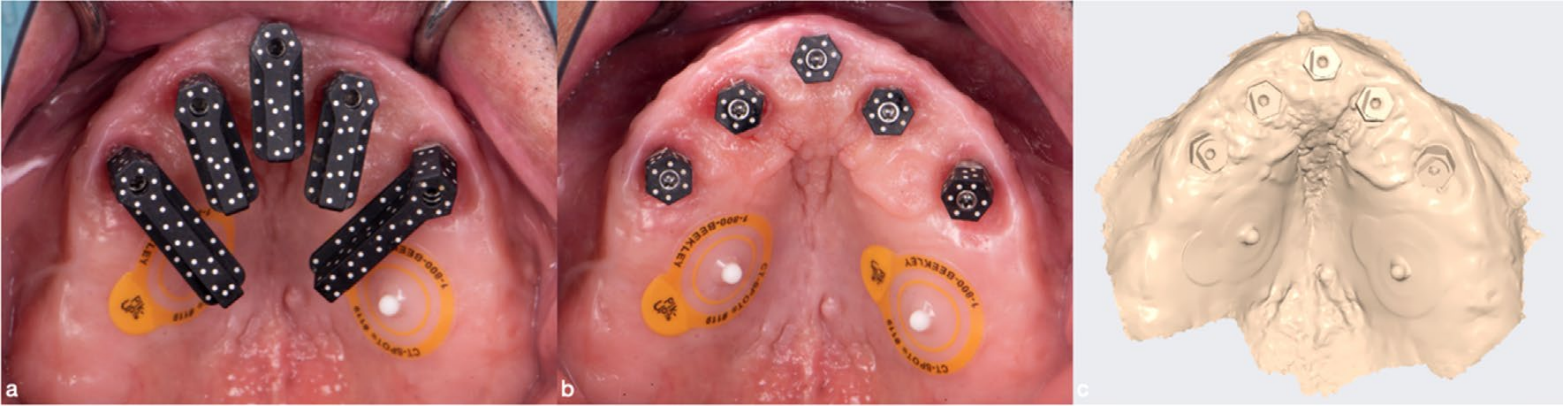
Intraoral data acquisition for prosthetic phase (a) clinical situation with coded scanbodies (Aoralscan Elite; Shining 3D Dental, Hangzhou, China) in situ for capturing the implant position, (b) clinical situation with CAP scanbodies (Aoralscan Elite; Shining 3D Dental, Hangzhou, China) in situ for digital impression to capture the soft tissues. The utilized system allows for the scan and alignment of the coded and CAP scanbodies the same device and systems, (c) intraoral scan.

**FIGURE 17:**
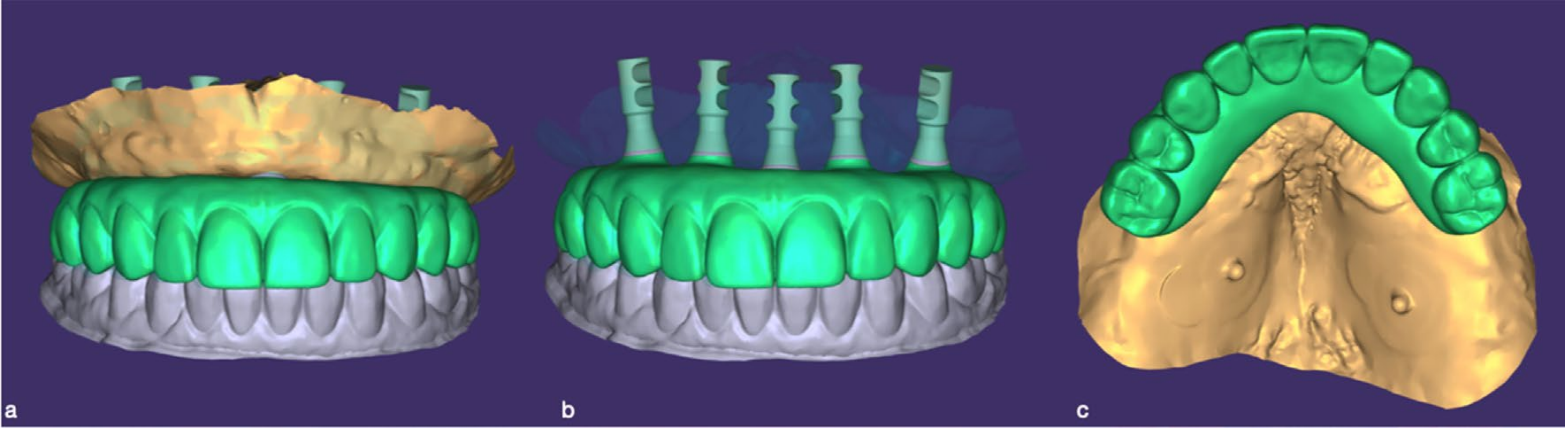
(a–c) Computer‐aided design of the implant‐supported prothesis.

**FIGURE 18:**
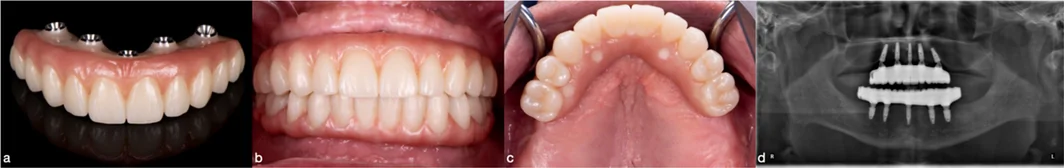
Delivery of the implant‐supported prosthesis (a) milled screw‐retained implant‐supported prosthesis on titanium abutments (Variobase; Straumann AG), (b, c) clinical situation after delivery of the iFDP, (d) final radiograph.

**FIGURE 19:**
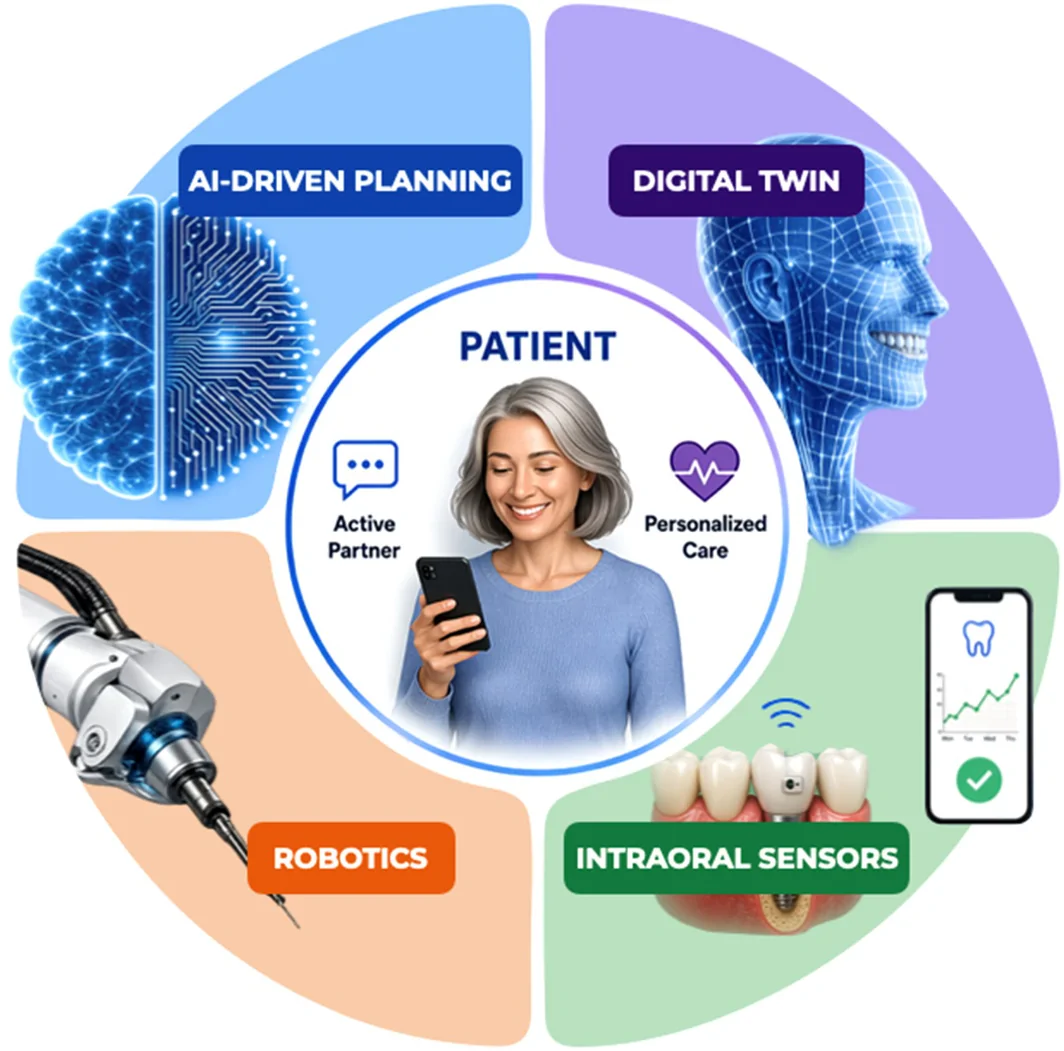
Graphical summary of the future digital ecosystem in implant dentistry.

## Disclosure

This manuscript used NLP (ChatGPT) for proofreading an entirely human‐generated text and for creating part of the illustrative figures, but no other use of AI was made.

## Data Availability

The data that support the findings of this study are available from the corresponding author upon reasonable request.
